# The incidence rates and prevalence of treated glaucoma in the United Kingdom, 2000–2022

**DOI:** 10.1371/journal.pone.0354463

**Published:** 2026-09-03

**Authors:** Luis Velez-Nandayapa, Laura Eggenschwiler, Susan S. Jick, Christoph R. Meier, Claudia Becker

**Affiliations:** 1 Basel Pharmacoepidemiology Unit, Division of Clinical Pharmacy and Epidemiology, Department of Pharmaceutical Sciences, University of Basel, Basel, Switzerland; 2 Hospital Pharmacy, University Hospital Basel, Basel, Switzerland; 3 Augenpraxis Kuessnacht, Kuessnacht, Switzerland; 4 Boston University School of Public Health, Boston, Massachusetts, United States of America; 5 Boston Collaborative Drug Surveillance Program, Lexington, Massachusetts, United States of America; Seirei Hamamatsu General Hospital, JAPAN

## Abstract

**Purpose:**

Quantify the incidence rates and prevalence of treated glaucoma in the United Kingdom (UK).

**Methods:**

Using data from the Clinical Practice Research Datalink (CPRD) GOLD, we calculated crude and age-standardised incidence rates and prevalence of treated glaucoma in adult patients from 2000 to 2022, stratifying by age, sex, year of diagnosis, and region.

**Results:**

We identified 112,632 cases of glaucoma in 82.7 million person-years (py) from 2000 to 2022.

The age-standardised incidence rates of glaucoma remained stable from 2000 to 2019 (128.77, 95% CI 125.15–132.40 to 112.63, 95% CI 108.75–116.52 per 100,000 py) with a marked reduction in 2020 (69.50, 95% CI 66.31–72.68 per 100,000 py) and a slight increase in 2021 and 2022 (94.09, 95% CI 90.23–97.96 to 91.70, 95% CI 87.61–95.79 per 100,000 py). Incidence rates sharply increased with age, peaking at 579.70 (95% CI 572.27–587.13 per 100,000 py) in the 80–89 age group, with more males in all age groups. The incidence rates of glaucoma were highest in England, London and Wales (3–8% above average), and lower in Northern Ireland and Scotland (11–16% below average).

The prevalence of glaucoma increased by 24.7% from 1.18% (95% CI 0.89–1.47%) to 1.47% (95% CI 1.03–1.90%). It was highest in the elderly >90 years (11.5%).

**Conclusions:**

In our large nationwide study in the UK, the incidence rates of glaucoma in the UK remained fairly stable from 2000 to 2019, with a significant decrease observed in 2020 due to the COVID-19 pandemic. Meanwhile, the prevalence of glaucoma has increased, reflecting the aging population. Both the incidence rates and prevalence varied widely across age groups, sexes, and different regions of the UK. Considering the aging UK population and the increasing prevalence of glaucoma, this eye condition represents a growing burden on the National Health Service in the UK.

## Introduction

Glaucoma refers to a group of diseases in which progressive optic nerve damage leads to vision loss [1–3]. It is classified as primary and secondary glaucoma (SG). Primary glaucoma is further subdivided into primary open angle glaucoma (POAG) and primary angle closure glaucoma (PACG) [1–3]. As the second leading cause of blindness worldwide [4], glaucoma accounts for about 10% of blindness registrations in the UK [5]. The World Health Organization (WHO) estimates that 80% of visual impairment is preventable or treatable, indicating that cost-effective interventions can alleviate the burden of visual impairment [6].

Researchers estimated that in 2010, 60.5 million people globally were affected by POAG and PACG, with projections suggesting a rise to 79.6 million by 2020 [4]. Glaucoma has become as a significant public health concern, prompting numerous population-based studies investigating its prevalence across various regions around the world. The variations in findings across these studies have led to the publication of several meta-analyses that pooled data to provide a more comprehensive understanding of the disease. Among the most representative meta-analyses, based on a broad global scope, one published in 2014 [7] included 50 studies involving 252,894 subjects, revealing an overall prevalence of POAG and PACG combined at 3.54% in patients 40–80 years old. Another meta-analysis in 2016 [8] examined 81 publications with a total of 216,214 participants, reporting a prevalence for POAG alone of 2.2%.

In the UK, previous estimates show a glaucoma prevalence of 2% to 4.2% among individuals aged 45 and older, affecting 11.2% of men and 10.8% of women in their 80s [9–12]. With the aging population, the number of individuals with glaucoma is expected to increase globally to 111.8 million by 2040 [7]. In the UK, projections indicate a 44% increase in glaucoma cases, an 18% rise in glaucoma suspects, and a 16% increase in ocular hypertension cases from 2015 to 2025 [13].

In contrast to numerous reports on the prevalence of glaucoma, population-based incidence studies are less common. A meta-analysis published in 2024 [14] yielded a global incidence rate of POAG at 234.6 cases per 100,000 person-years (py) among those aged 40–79 years.

Studies from Scandinavian countries using prescriptions or combined approach of prescriptions, glaucoma diagnosis and surgeries as a proxy to estimate the prevalence and incidence of glaucoma provide estimates of glaucoma prevalence ranging from 0.76% to 2.19% for individuals over 40, with prevalence reaching between 8% and 11% in those over 80 years old [15–19].

In the UK, the Royal Colleague of Ophthalmologists (RCOphth) and the National Institute for Health and Care Excellence (NICE) published seven major guidelines between 1997 and 2022 for primary eye care professionals for the management of glaucoma [20–26]. These guidelines detail case definitions for IOH, POAG and PACG, risk factors, diagnostic criteria, and treatment recommendations.

Given the rapid increase in global aging populations, accurate projections of glaucoma prevalence and incidence are vital for creating effective health policies and screening strategies [7]. The only proven intervention for preventing and controlling glaucoma is lowering intraocular pressure (IOP), which has been shown to reduce glaucoma progression in a variety of large-scale clinical trials [27–29]. Reduction of intraocular pressure is achieved by drug treatment or surgery including laser therapy and Minimally Invasive Glaucoma Surgery (MIGS) [2,3].

To our knowledge, treatment rates and prevalence for treated glaucoma in a large UK sample have not been quantified. Thus, we estimated the incidence of new treatments and treatment prevalence for glaucoma using data from the UK Clinical Practice Research Datalink (CPRD) to inform public health planning.

## Methods

### Data source

We performed a retrospective, longitudinal cohort study in a national electronic health record database, the UK CPRD GOLD. This database contains anonymized medical records from over 600 general practices and more than 15 million patients (at the time of the study period) who are representative of the UK population with regard to age, sex, and ethnicity [30]. Information is recorded by general practitioners (GPs) as part of routine patient care, and includes patient demographics, symptoms, diagnostic tests, diagnoses, drug therapies, health-related behaviour, and referrals to secondary care [30]. General Practitioners generate drug prescriptions directly with the computer using a coded drug dictionary. The database has been described in detail [31,32] and has been validated extensively [33–35]. The data utilized in this retrospective study were accessed for research purposes on October 7th, 2024. To ensure the confidentiality and anonymity of the participants, it is important to note that the authors did not have access to any information capable of identifying individual participants during the data collection process, nor did they have such access after the completion of data gathering.

### Population

The base population consisted of all adult patients (≥18 years-old) with an active record in the CPRD GOLD between January 2000 and December 2022. We defined the start of follow up as the date when a patient turned 18 years old, or the date when the patient was registered with the GP, whichever came later. We excluded individuals with a record of congenital or childhood glaucoma from the study population, since we were interested in assessing the incidence rates and prevalence of glaucoma. We also excluded patients with bilateral blindness before the start of follow-up. Finally, we excluded patients with less than three years of medical history on the database before the start of follow up to be more confident that cases were incident.

### Case definition

We estimate glaucoma cases using prescription data and/or glaucoma surgeries as proxies. A case was defined as someone who newly received treatment for glaucoma, consisting of either two or more glaucoma-related prescriptions and/or a recorded glaucoma surgery during the study period. Many authors in Scandinavian countries have employed this approach to estimate glaucoma cases. For example, a 1989 study across Scandinavian nations [16], a 1986 Icelandic study [15], a 2015 study in Denmark [17,19], and a 2020 Norwegian study [18] all utilized this method to estimate glaucoma cases.

We considered substances included in section “S01E Antiglaucoma preparation and miotics” from the WHO ATC list as relevant therapeutics. Topical antiglaucoma drugs include prostaglandin analogues, carbonic anhydrase inhibitors, beta-receptor antagonists, adrenergic agonists, parasympathomimetics and Rho kinase inhibitors [3].

We defined the index date as the earliest date of the recorded prescription or surgery for glaucoma.

### Statistical analysis

We calculated incidence rates and prevalence of treated glaucoma from 2000 to 2022, stratified by age, sex, year of diagnosis and UK region: England (excluding London), London, Scotland, Wales, and Northern Ireland.

To derive crude incidence rates, we divided the number of new glaucoma cases during the study period by the total number of person-years at risk. We accumulated person-years between the start of the study period and the end of follow-up (i.e., the date the patient had a first treatment for glaucoma, left the practice, had a diagnosis of bilateral blindness, died, or the study period ended, whichever came first). We also computed age-standardised incidence rates of glaucoma using the European Standard Population 2010 [36]. We additionally assessed the prevalence as the number of glaucoma cases divided by the number of subjects in the base population in any given stratum. We performed all analyses using SAS, version 9.4.

### Ethics statement

This retrospective study is based in part on data from the Clinical Practice Research Datalink (CPRD) obtained under license from the UK Medicines and Healthcare Products Regulatory Agency. The data is provided by patients and collected by the NHS as part of their care and support. The interpretation and conclusions contained in this study are those of the authors alone. This study was approved by the Independent Scientific Advisory Committee (ISAC) for Medicines and Healthcare Products Regulatory Agency (MHRA) database research (Protocol no: 17_006), and it is compliant with the requirements of the Declaration of Helsinki. The study protocol was made available to reviewers upon request. Copyright © 2022, re-used with the permission of The Health & Social Care Information Centre. All rights reserved.

## Results

**Fig 1:**
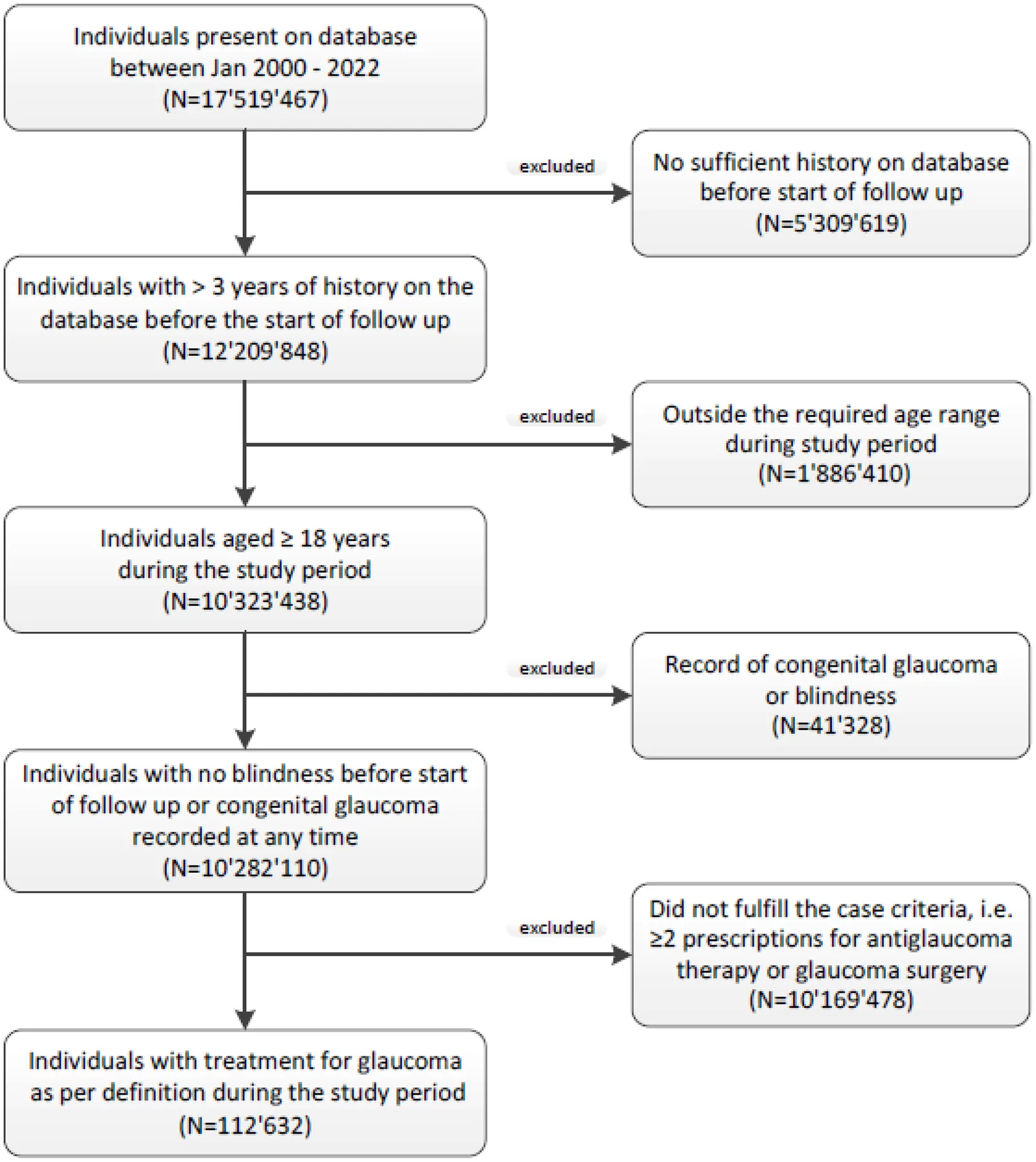
Flowchart of patients identified with treatment for glaucoma in the UK.

We identified 112,632 cases of glaucoma who met our study definition in 82.7 million py of follow-up from 2000 to 2022. A little more than half of the cases were female (51.9%). The majority of patients received only pharmacological treatment for glaucoma (approximately 88%), around 4% had surgery for glaucoma with no pharmacological treatment, and around 8% received both. Fig 1 shows the process of selecting the patients identified with treatment for glaucoma.

**Fig 2:**
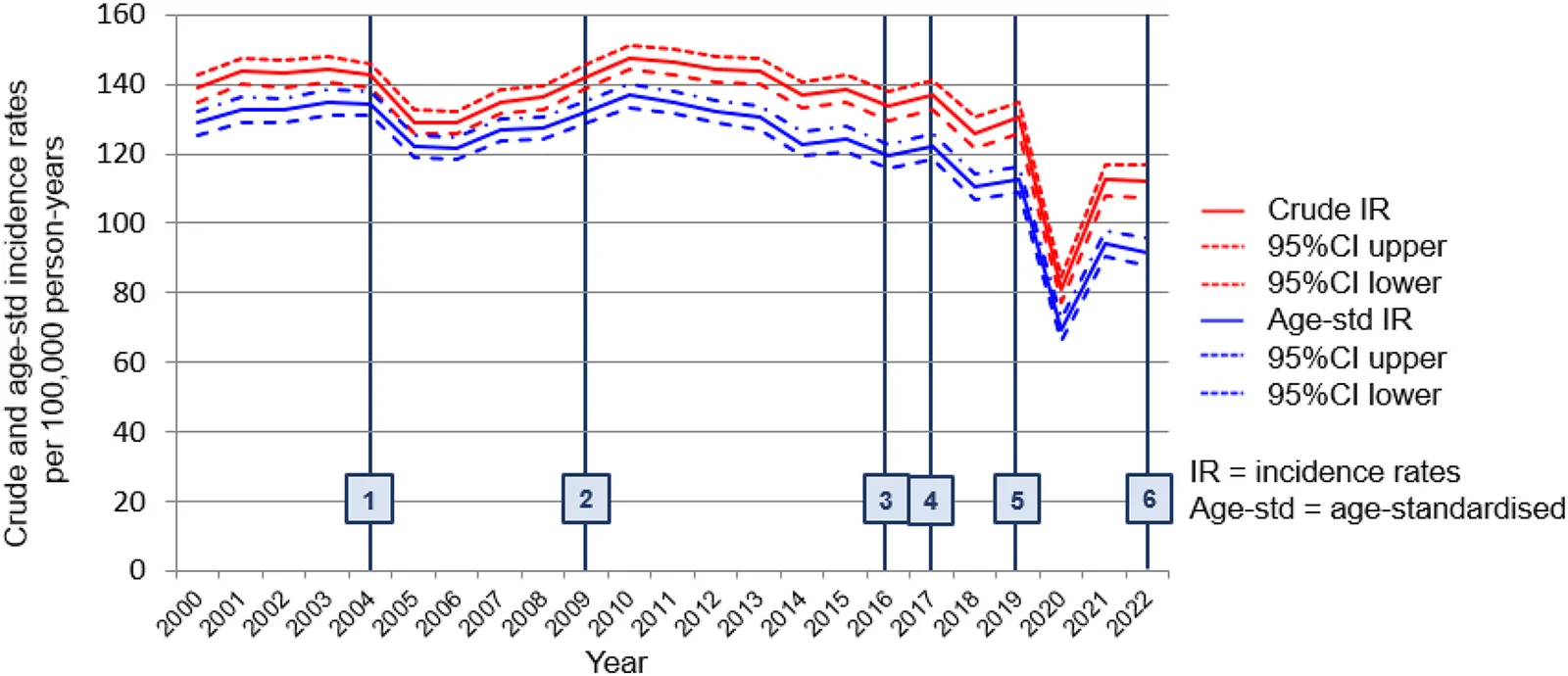
Crude and age-standardised incidence rates of glaucoma [per 100,000 person-years and 95% CI] in the UK from 2000 to 2022; dotted lines represent the upper and lower limits of 95% confidence intervals. RCOphth: Royal College of Ophthalmologists; NICE: National Institute for Health and Care Excellence; 1. RCOphth, Guidelines for the management of open angle glaucoma and ocular hypertension (2004); 2. NICE, Glaucoma: diagnosis and management (2009); 3. RCOphth, Commissioning Guide: Glaucoma (2016); 4. NICE (guideline NG81), Glaucoma: diagnosis and management (2017); 5. NICE, Exceptional surveillance of glaucoma: diagnosis and management (2019); 6. RCOphth, Guidelines for the management of angle-closure glaucoma (2022).

The average crude and age-standardised incidence rates (95% CI) of glaucoma in the UK over the full study period were 136.13, 95% CI 135.33–136.92, and 124.24, 95% CI 123.52–124.97 per 100,000 py, respectively. The incidence rates, age-standardised of glaucoma, remained stable from 2000 to 2019 (128.77, 95% CI 125.15–132.40 to 112.63, 95% CI 108.75–116.52) with a marked reduction in 2020 (69.50, 95% CI 66.31–72.68) and a slight increase in 2021 and 2022 (94.09, 95% CI 90.23–97.96 to 91.70, 95% CI 87.61–95.79 per 100,000 py, Fig 2).

**Fig 3:**
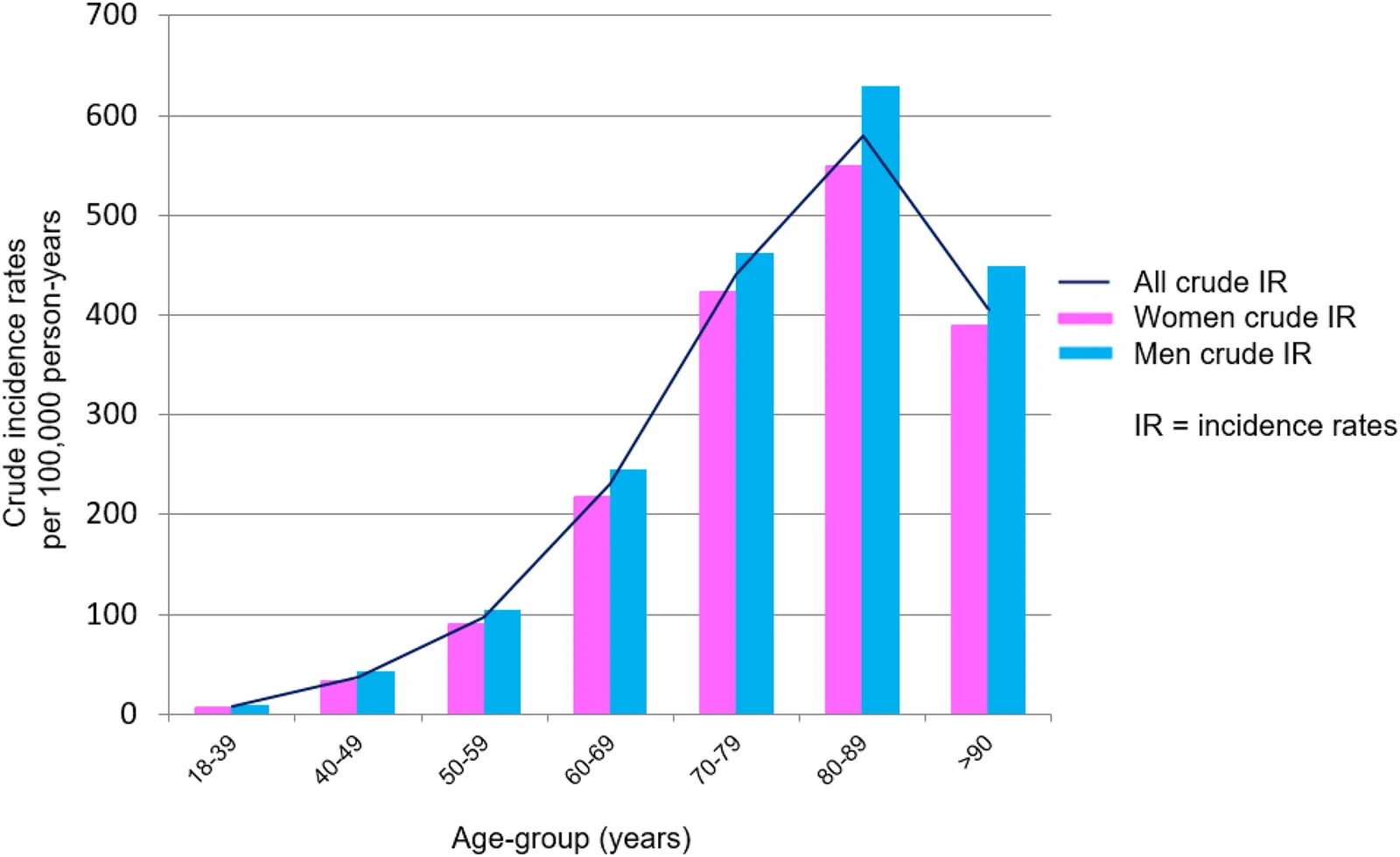
Crude incidence rates [per 100,000 person-years] of glaucoma in the UK by age-group and sex from 2000 to 2022. RCOphth: Royal College of Ophthalmologists; NICE: National Institute for Health and Care Excellence; 1. RCOphth, Guidelines for the management of open angle glaucoma and ocular hypertension (2004); 2. NICE, Glaucoma: diagnosis and management (2009); 3. RCOphth, Commissioning Guide: Glaucoma (2016); 4. NICE (guideline NG81), Glaucoma: diagnosis and management (2017); 5. NICE, Exceptional surveillance of glaucoma: diagnosis and management (2019); 6. RCOphth, Guidelines for the management of angle-closure glaucoma (2022).

However, the incidence rates of glaucoma sharply increased with increasing age. The incidence rates were highest in the age-group 80–89 years: with an average crude rate of 579.70, 95% CI 572.29–587.11 per 100,000 py over the time of the study period (Fig 3).

The incidence rates of glaucoma were almost evenly distributed among both sexes up to the age of 59 years, thereafter male cases prevailed more clearly (Fig 3).

**Fig 4:**
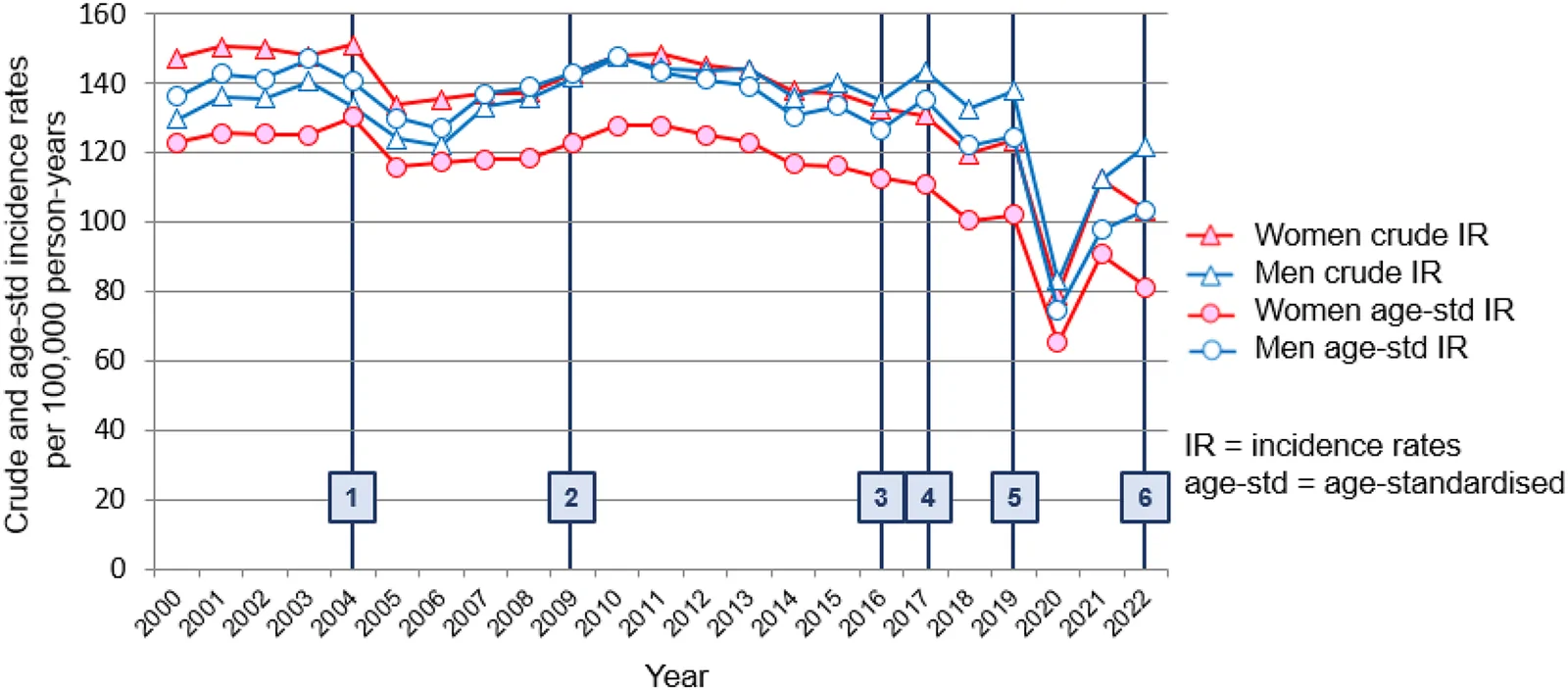
Crude and age-standardised incidence rates [per 100,000 person-years] of glaucoma in the UK by sex from 2000 to 2022. RCOphth: Royal College of Ophthalmologists; NICE: National Institute for Health and Care Excellence; 1. RCOphth, Guidelines for the management of open angle glaucoma and ocular hypertension (2004); 2. NICE, Glaucoma: diagnosis and management (2009); 3. RCOphth, Commissioning Guide: Glaucoma (2016); 4. NICE (guideline NG81), Glaucoma: diagnosis and management (2017); 5. NICE, Exceptional surveillance of glaucoma: diagnosis and management (2019); 6. RCOphth, Guidelines for the management of angle-closure glaucoma (2022).

Additionally, in our analysis by sex (Fig 4), we observed higher incidence rates (age-standardised) in male vs female patients from the year 2000–2022. With rates in 2000 of 136.08, 95% CI 130.29–141.88 vs 122.97, 95% CI 118.29–127.66 per 100,000 py respectively and incidence rates in 2022 of 103.53, 95% CI 97.11–109.94 vs 81.32, 95% CI 76.08–86.56 per 100,000 py respectively.

**Fig 5:**
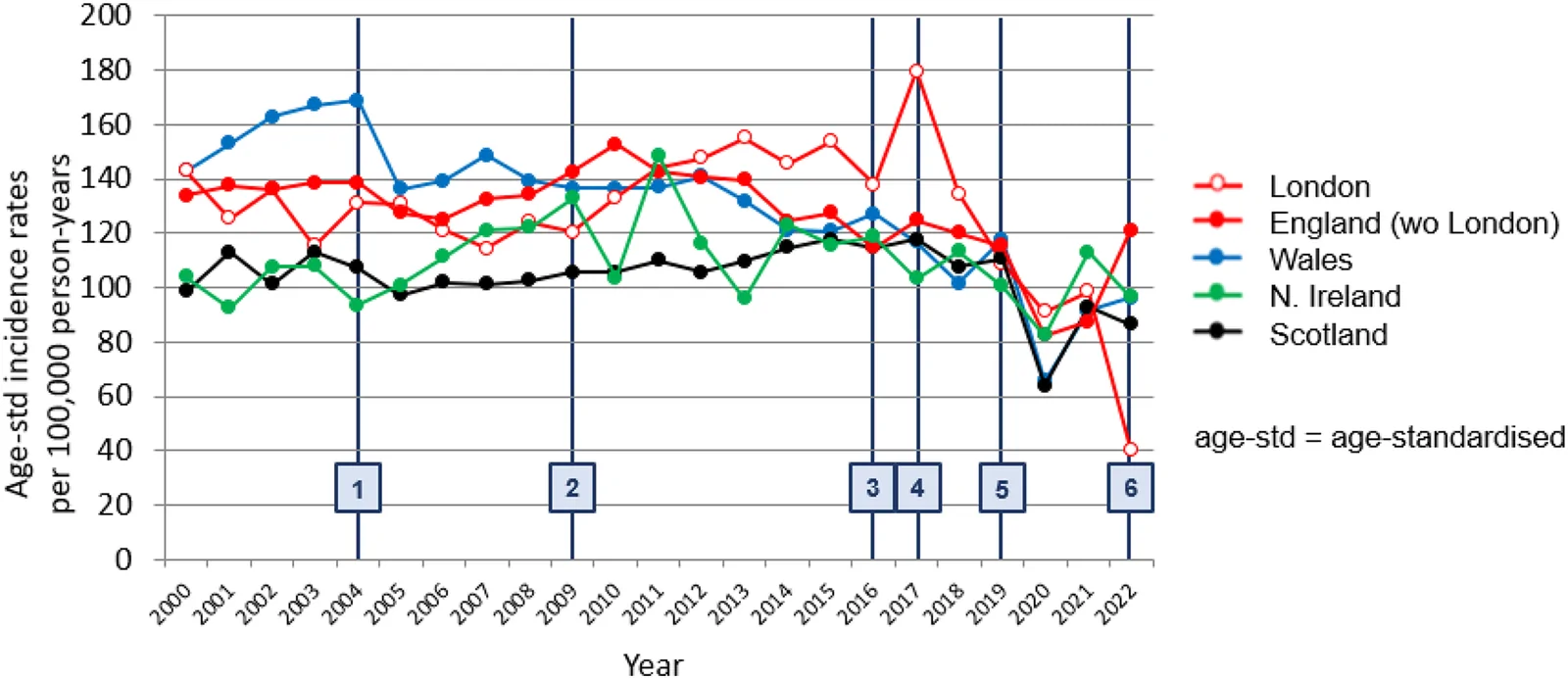
Age-standardised incidence rates [per 100,000 person-years] of glaucoma in the UK by region from 2000 to 2022. RCOphth: Royal College of Ophthalmologists; NICE: National Institute for Health and Care Excellence; 1. RCOphth, Guidelines for the management of open angle glaucoma and ocular hypertension (2004); 2. NICE, Glaucoma: diagnosis and management (2009); 3. RCOphth, Commissioning Guide: Glaucoma (2016); 4. NICE (guideline NG81), Glaucoma: diagnosis and management (2017); 5. NICE, Exceptional surveillance of glaucoma: diagnosis and management (2019); 6. RCOphth, Guidelines for the management of angle-closure glaucoma (2022).

In our analysis by region, we observed the highest age-standardised incidence rates of glaucoma in England without London (average of 134.16, 95% CI 133.04–135.29 per 100,000 py), followed by London (average of 133.71, 95% CI 130.85–136.57 per 100,000 py) and Wales (average of 128.02, 95% CI 126.28–129.75 per 100,000 py). The lowest age-standardised incidence rates of glaucoma were in Northern Ireland (average of 110.05, 95% CI 106.95–113.14 per 100,000 py) and Scotland (average 104.07, 95% CI 102.74–105.39 per 100,000 py, Fig 5).

The incidence rates of glaucoma decreased in all regions in the year 2020, the beginning of the pandemic. The only region that returned to almost pre-pandemic treatment levels after 2020 was England without London.

### The prevalence of glaucoma

In 2000, the age-standardised prevalence of glaucoma was 1.18, 95% CI 0.89–1.47 compared to 1.47, 95% CI 1.03–1.90 in 2022 (S1 Table). Thus, the prevalence of glaucoma in the UK increased by 24.7% during the study period. The prevalence was higher in the elderly and affected 9.4% of the population of those over 80 years of age but was only present in 0.3% of the population aged 18–59 years. The prevalence of glaucoma was highest (11.5%) in people over the age of 90 years (S2 Table). As already observed with the incidence rates, the prevalence of glaucoma was higher in males than females (S3 Table).

**Fig 6:**
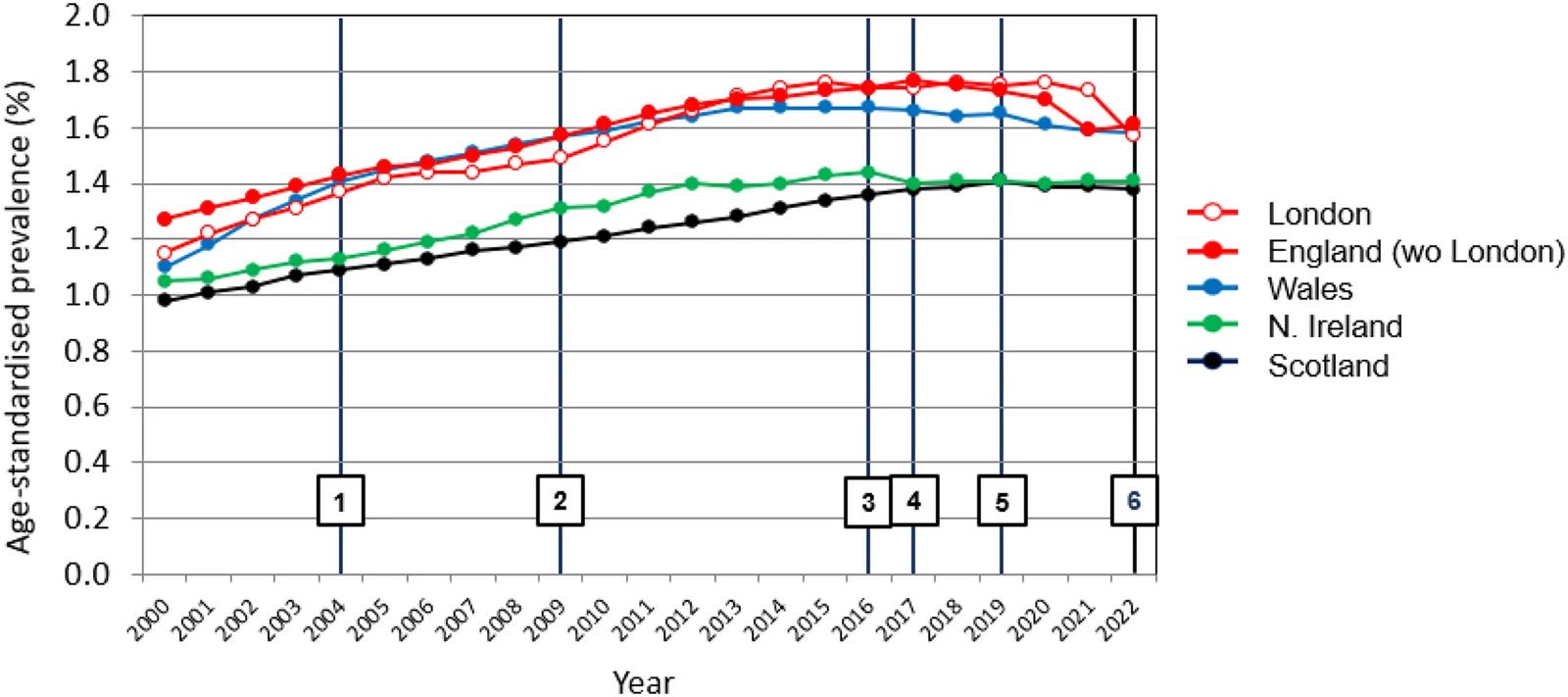
Age-standardised prevalence of glaucoma (95%CI) in the UK by region from 2000 to 2022. RCOphth: Royal College of Ophthalmologists; NICE: National Institute for Health and Care Excellence; 1. RCOphth, Guidelines for the management of open angle glaucoma and ocular hypertension (2004); 2. NICE, Glaucoma: diagnosis and management (2009); 3. RCOphth, Commissioning Guide: Glaucoma (2016); 4. NICE (guideline NG81), Glaucoma: diagnosis and management (2017); 5. NICE, Exceptional surveillance of glaucoma: diagnosis and management (2019); 6. RCOphth, Guidelines for the management of angle-closure glaucoma (2022).

In our analysis by region (Fig 6), we noted an increase in the prevalence of glaucoma across all areas of the UK. The highest observed increase in prevalence from 2000 to 2022 was in Wales, showing an increase of 43.5%. This was followed by Scotland with a 41.8% increase, London with 36.4%, and Northern Ireland with 34.4%. England had the lowest increase with 26.6%, but the highest absolute prevalence. We observed a slight decrease in the prevalence of glaucoma in three regions (London, England, and Wales) during the years 2019–2022, while the prevalence in Northern Ireland and Scotland remained steady during the same period.

## Discussion

In our large observational study using primary care data from the UK, we observed relatively steady incidence rates of treated glaucoma over a 20-year period (2000–2019). Towards the end of the study period, there was an abrupt decrease in incidence rates of glaucoma starting in 2020, coinciding with the COVID-19 pandemic, which was declared a Public Health Emergency by the WHO on January 30, 2020 [37]. In the UK, significant restrictions related to the pandemic were introduced on March 23, 2020 [38]. Coinciding with the lockdown, we observed a reduction of 38.3% in incidence rates of glaucoma in 2020 (69.50, 95% CI 66.31–72.68 cases per 100,000 py) compared to 2019 (112.63, 95% CI 108.75–116.52 cases per 100,000 py).

In contrast to the stable incidence rates of glaucoma observed from 2000 to 2019, we recorded a 24.7% increase in the prevalence of glaucoma over the time of our study. There was a steady increase in the prevalence from the year 2000–2015, followed by a constant trend from 2016 to 2019 and a slight decrease from 2020 to 2022.

We reported an overall age-standardised prevalence of glaucoma of 1.46%, with a prevalence of 1.72% in those over 40–79 years old and of 9.40% in those over 80 years. Similar findings have been observed in other studies. A meta-analysis published in 2014 [7] yielded an overall prevalence of POAG and PACG combined at 3.54% in patients aged 40–80 years, and a prevalence of 2.93% in Europe. Another meta-analysis from 2016 [8] reported an overall prevalence of POAG alone of 2.2%. A study in Scandinavian countries published in 1989 [16] using prescriptions as a proxy to estimate the prevalence of glaucoma, reported slightly lower overall prevalences of 0.33% to 0.92% across countries. The prevalence was higher in those over 40 years: 1.44% to 3.15%. Another study from Iceland published in 1986 [15] reported an overall prevalence of 0.83%, 3.53% in those over 50 years and 10.75% in those over 80 years, also quite similar to our results.

Prevalence rates have been reported to increase steadily in Denmark [17,19], while constant prevalences over a 15-year period have been reported in a study in Norway in 2020 [18]. Our results indicate an increase in prevalence from 2000 to 2015 (possibly attributed to the aging population in the UK), followed by a stable trend from 2016 to 2019, and a slight decrease from 2020 to 2022, coinciding with the COVID-19 pandemic (S1 Fig).

Our findings are also aligned with more recent epidemiological studies using glaucoma prescriptions as a proxy for glaucoma cases [17,18], reporting constant incidence rates (S2 Fig).

We reported a steady trend in incidence rates of glaucoma from 2000 to 2010; however, a slight decrease in these incidence rates was observed from 2011 to 2019. The publication of three significant guidelines during this period – one from The Royal College of Ophthalmologists [22] and two from the National Institute for Health and Care Excellence (NICE) [25,26] may have contributed to this slight decline. These guidelines offer updated recommendations and protocols for the management and treatment of glaucoma, which could enhance the adoption of best practices among healthcare providers. Consequently, while the new treatment rates have experienced a minor decrease, the dissemination of this guidance highlights the ongoing commitment to optimizing patient care and outcomes.

While glaucoma can affect people of all ages, it predominantly impacts the elderly [39]. In our study, the incidence rates of glaucoma increased with age and peaked in the 80–89-year age group. The prevalence of glaucoma was highest among those over 90 years old, reaching 11.5%. Our findings are in line with those observed in meta-analyses [7,8] and with those of previous epidemiological studies using glaucoma prescriptions as a proxy for glaucoma cases in Scandinavian countries [15–19]. In general, these observations align with projections from systematic review and meta-analyses indicating that, due to the increase in aging populations, the number of people with glaucoma is expected to rise substantially in the next 20 years [7,8].

There is no clear consensus in the literature regarding the association between sex and glaucoma [40]. Some studies suggest that women are at higher risk for primary angle-closure glaucoma (PACG), likely due to anatomical predisposition [41]. Female sex has also been identified as a risk factor for normal tension glaucoma [42]. The Rotterdam Study demonstrated an increased risk of primary open-angle glaucoma (POAG) in women who experienced early menopause [43]. A study from Denmark [17] reported higher rates of both prevalence and incidence for females vs males. In our study, the increase in prevalence among females (27.0%) was greater than that among males (21.2%) from 2000 to 2022.

In contrast, we report for both the incidence rates and prevalence of glaucoma higher rates for males than females. This is in line with the results from two meta-analyses published in 2014 [7] and in 2016 [8]: Both found that men were more likely to have POAG than women. A study in Iceland in 1986 [15] also yielded a higher prevalence in males vs females, and a study in Norway in 2020 [18] found higher incidence rates in males vs females, and higher prevalences for males from 0–69 years old and >90 years.

Interestingly, we observed differences in the incidence rates of glaucoma between the various UK regions in our study. We noted the highest incidence rates in London, followed by the rest of England and Wales, while Northern Ireland and Scotland had lower incidence rates of glaucoma with similar observations for the prevalence. The differences observed across regions may be explained by varying access to healthcare. In the UK, 84% of the population lives in urban areas, while 16% resides in rural areas. Regarding access to primary care, 94.2% of individuals in urban areas live within a 20-minute walk of a General Practitioner office, compared to only 19.4% of those in rural areas in England [44]. This disparity could account for the prevalence of glaucoma observed in our study until 2019 (pre-pandemic levels), where urban areas such as London (98% urban) and the rest of England (83% urban) exhibited the highest prevalence of glaucoma, at 1.75% and 1.73% respectively. In contrast, Wales, Northern Ireland, and Scotland had prevalences of 1.65%, 1.41%, and 1.41%, respectively.

The meta-analysis from 2014 [7], found that people living in urban areas were 58% more likely to receive a diagnosis of primary open-angle glaucoma (POAG) than those living in rural areas. Similarly, the study in Denmark in 2015 [17] reported a significantly higher prevalence of glaucoma in the capital region of Denmark (6.28%, 95% CI 6.21–6.36) compared to other regions, such as the Northern Denmark Region (3.96%, 95% CI 3.86–4.06). Aside from the explanation of easier access to healthcare in urban areas, other potential differences between a rural and an urban lifestyle might contribute to differences in glaucoma prevalence, such as stress, pollution, diet, physical activity, and comorbid diseases [7]. Further studies are needed to explore the mechanisms underlying these differences in prevalence by residential area.

The development and periodic updating of clinical guidelines by the Royal College of Ophthalmologists and the National Institute for Health and Care Excellence have played a crucial role in shaping the management of glaucoma and intraocular hypertension (IOH) in the UK during the last twenty years. The seven major guidelines published between 1997 and 2022 provide comprehensive frameworks, including case definitions for IOH, primary open-angle glaucoma (POAG), and primary angle-closure glaucoma (PACG), as well as identified risk factors, diagnostic criteria, and evidence-based treatment recommendations. These guidelines have supported standardised clinical practice across primary and specialised eye care providers, facilitating early detection, consistent diagnosis, and appropriate management strategies. However, the guidelines on the diagnosis and management of glaucoma in the UK during the period from 2000 to 2019 (covering 20 years in the pre-pandemic era) do not appear to have had a significant effect on the incidence rates and prevalence of glaucoma in the UK in our study.

This study has several strengths. To the best of our knowledge, it represents the largest nationwide study of incidence rates and prevalence of glaucoma in the UK.

The data source is a well-established primary care database known for its high quality, completeness, and representativeness of the UK population. Epidemiological studies using large databases are cost-effective and likely provide valid population-based data on the epidemiology of glaucoma. However, our study also has some limitations.

Our approach, as well as that of other authors [15–19], to estimate glaucoma cases using prescription data and/or glaucoma surgeries as a proxy, must not be compared to true glaucoma incidence or prevalence of glaucoma (i.e., assessed through surveys and including an ophthalmologic examination). First, our rates include patients with glaucoma as well patients with intraocular hypertension without glaucoma. Second, it is likely that a considerable number of glaucoma cases remain undiagnosed and thus untreated, especially if patients did not seek medical attention for diagnostic screening

## Conclusions

The incidence rates of glaucoma in the UK remained fairly stable from 2000 to 2019, with a significant decrease observed in 2020 due to the COVID-19 pandemic. Meanwhile, the prevalence of glaucoma has increased, reflecting the aging population. Both the incidence rates and prevalence of glaucoma varied widely across age groups, sexes, and different regions of the UK. Considering the aging UK population and the increasing treatment prevalence of glaucoma, this eye condition represents a growing burden on the National Health Service in the UK.

## Supporting information

S1 Table Crude and age-standardised prevalence of glaucoma (95% CI) in the UK from 2000 to 2022. (PDF)

S2 Table Crude prevalence of glaucoma (95% CI) in the UK from 2000 to 2022 by age-group. (PDF)

S3 Table Crude and age-standardised prevalence of glaucoma (95% CI) in the UK from 2000 to 2022 by sex. (PDF)

S1 Fig Prevalence of glaucoma from different studies. (PDF)

S2 Fig Incidence rates of glaucoma [per 100,000 person-years] from different studies. (PDF)
