# Supplementary material for: The incidence rates and prevalence of treated glaucoma in the United Kingdom, 2000–2022
Source: PLoS One. 2026 Sep 3;21(9):e0354463. doi: 10.1371/journal.pone.0354463 (PMC13541118; doi:10.1371/journal.pone.0354463)
Supplement: Supplementary file 1 [file pone.0354463.s001.pdf]

S1 Table. Crude and age-standardised prevalence of glaucoma (95% CI) in the UK from 2000 to 2022.

Year	Crude prevalence % (95% CI)	Age-standardised prevalence % (95% CI)
2000	1.23 (1.22-1.24)	1.18 (0.89-1.47)
2001	1.27 (1.26-1.28)	1.22 (0.93-1.51)
2002	1.31 (1.30-1.32)	1.26 (0.97-1.55)
2003	1.36 (1.35-1.37)	1.31 (1.01-1.60)
2004	1.40 (1.39-1.40)	1.35 (1.05-1.64)
2005	1.43 (1.42-1.44)	1.38 (1.08-1.67)
2006	1.45 (1.44-1.46)	1.40 (1.10-1.69)
2007	1.49 (1.48-1.50)	1.42 (1.13-1.72)
2008	1.52 (1.51-1.53)	1.45 (1.15-1.75)
2009	1.56 (1.55-1.57)	1.48 (1.18-1.78)
2010	1.61 (1.60-1.62)	1.51 (1.21-1.82)
2011	1.65 (1.64-1.66)	1.54 (1.23-1.85)
2012	1.70 (1.69-1.71)	1.57 (1.26-1.89)
2013	1.74 (1.73-1.75)	1.59 (1.28-1.91)
2014	1.77 (1.76-1.78)	1.60 (1.27-1.92)
2015	1.79 (1.78-1.80)	1.60 (1.26-1.94)
2016	1.80 (1.79-1.81)	1.59 (1.23-1.96)
2017	1.78 (1.77-1.79)	1.58 (1.20-1.97)
2018	1.78 (1.77-1.79)	1.57 (1.17-1.96)
2019	1.80 (1.79-1.82)	1.56 (1.16-1.96)
2020	1.80 (1.78-1.81)	1.52 (1.12-1.93)
2021	1.76 (1.74-1.77)	1.48 (1.06-1.90)
2022	1.80 (1.79-1.82)	1.47 (1.03-1.90)

CI: Confidence Intervals
