# Supplementary material for: The incidence rates and prevalence of treated glaucoma in the United Kingdom, 2000–2022
Source: PLoS One. 2026 Sep 3;21(9):e0354463. doi: 10.1371/journal.pone.0354463 (PMC13541118; doi:10.1371/journal.pone.0354463)
Supplement: Supplementary file 2 [file pone.0354463.s002.pdf]

S2 Table. Crude prevalence of glaucoma (95% CI) in the UK from 2000 to 2022 by age-group.

Age-group	Males Crude prevalence % (95%CI)	Females Crude prevalence % (95%CI)	ALL Crude prevalence % (95%CI)
18-39	0.08 (0.08 - 0.08)	0.05 (0.05-0.05)	0.06 (0.06-0.07)
40-49	0.35 (0.34 - 0.35)	0.24 (0.24-0.25)	0.30 (0.29-0.30)
50-59	1.00 (1.00 - 1.01)	0.79 (0.78-0.79)	0.90 (0.89-0.90)
60-69	2.47 (2.46 - 2.48)	2.10 (2.09-2.11)	2.28 (2.27-2.29)
70-79	5.42 (5.39 - 5.44)	4.71 (4.69-4.73)	5.04 (5.02-5.05)
80-89	9.79 (9.75 - 9.83)	8.35 (8.32-8.38)	8.91 (8.89-8.93)
≥90	13.08 (12.97 - 13.20)	10.87 (10.80-10.93)	11.47 (11.41-11.52)
