# Supplementary material for: The incidence rates and prevalence of treated glaucoma in the United Kingdom, 2000–2022
Source: PLoS One. 2026 Sep 3;21(9):e0354463. doi: 10.1371/journal.pone.0354463 (PMC13541118; doi:10.1371/journal.pone.0354463)
Supplement: Supplementary file 3 [file pone.0354463.s003.pdf]

S3 Table. Crude and age-standardised prevalence of glaucoma (95% CI) in the UK from 2000 to 2022 by sex.

Year	Crude prevalence % (95%CI)		Age-standardised prevalence % (95%CI)	
	Males	Females	Males	Females
2000	1.21 (1.19-1.22)	1.25 (1.24-1.26)	1.32 (0.85-1.80)	1.06 (0.70-1.43)
2001	1.24 (1.23-1.26)	1.30 (1.28-1.31)	1.37 (0.89-1.84)	1.10 (0.74-1.47)
2002	1.27 (1.26-1.29)	1.35 (1.34-1.36)	1.41 (0.93-1.88)	1.15 (0.78-1.52)
2003	1.31 (1.30-1.33)	1.40 (1.38-1.41)	1.45 (0.98-1.92)	1.19 (0.82-1.56)
2004	1.34 (1.33-1.36)	1.44 (1.43-1.46)	1.49 (1.02-1.96)	1.23 (0.86-1.61)
2005	1.37 (1.36-1.39)	1.48 (1.46-1.49)	1.52 (1.05-1.99)	1.27 (0.89-1.64)
2006	1.40 (1.39-1.41)	1.50 (1.49-1.52)	1.54 (1.07-2.00)	1.29 (0.91-1.66)
2007	1.44 (1.42-1.45)	1.54 (1.52-1.55)	1.56 (1.09-2.03)	1.31 (0.93-1.69)
2008	1.48 (1.46-1.49)	1.57 (1.56-1.58)	1.59 (1.12-2.06)	1.34 (0.95-1.72)
2009	1.52 (1.50-1.53)	1.61 (1.59-1.62)	1.62 (1.14-2.09)	1.37 (0.98-1.76)
2010	1.57 (1.55-1.58)	1.65 (1.63-1.66)	1.65 (1.17-2.13)	1.40 (1.00-1.79)
2011	1.62 (1.60-1.63)	1.69 (1.67-1.70)	1.68 (1.20-2.16)	1.43 (1.03-1.83)
2012	1.67 (1.65-1.68)	1.73 (1.71-1.74)	1.71 (1.22-2.20)	1.46 (1.05-1.87)
2013	1.72 (1.71-1.74)	1.76 (1.74-1.77)	1.73 (1.24-2.23)	1.48 (1.06-1.89)
2014	1.76 (1.74-1.78)	1.78 (1.77-1.80)	1.73 (1.23-2.24)	1.48 (1.06-1.91)
2015	1.78 (1.76-1.80)	1.80 (1.79-1.82)	1.73 (1.21-2.26)	1.49 (1.04-1.94)
2016	1.80 (1.78-1.82)	1.80 (1.79-1.82)	1.72 (1.16-2.28)	1.48 (1.00-1.96)
2017	1.79 (1.77-1.81)	1.77 (1.75-1.79)	1.71 (1.12-2.30)	1.47 (0.97-1.98)
2018	1.80 (1.78-1.82)	1.76 (1.74-1.78)	1.69 (1.09-2.30)	1.46 (0.94-1.98)
2019	1.84 (1.82-1.86)	1.77 (1.75-1.79)	1.69 (1.08-2.30)	1.45 (0.93-1.97)
2020	1.84 (1.82-1.86)	1.76 (1.74-1.78)	1.66 (1.03-2.28)	1.41 (0.87-1.95)
2021	1.80 (1.77-1.82)	1.72 (1.70-1.74)	1.61 (0.97-2.25)	1.37 (0.82-1.92)
2022	1.87 (1.85-1.90)	1.74 (1.72-1.77)	1.60 (0.94-2.27)	1.35 (0.78-1.92)

CI: Confidence Intervals
