# Supplementary material for: The incidence rates and prevalence of treated glaucoma in the United Kingdom, 2000–2022
Source: PLoS One. 2026 Sep 3;21(9):e0354463. doi: 10.1371/journal.pone.0354463 (PMC13541118; doi:10.1371/journal.pone.0354463)

S1 Fig. Prevalence of glaucoma from different studies.

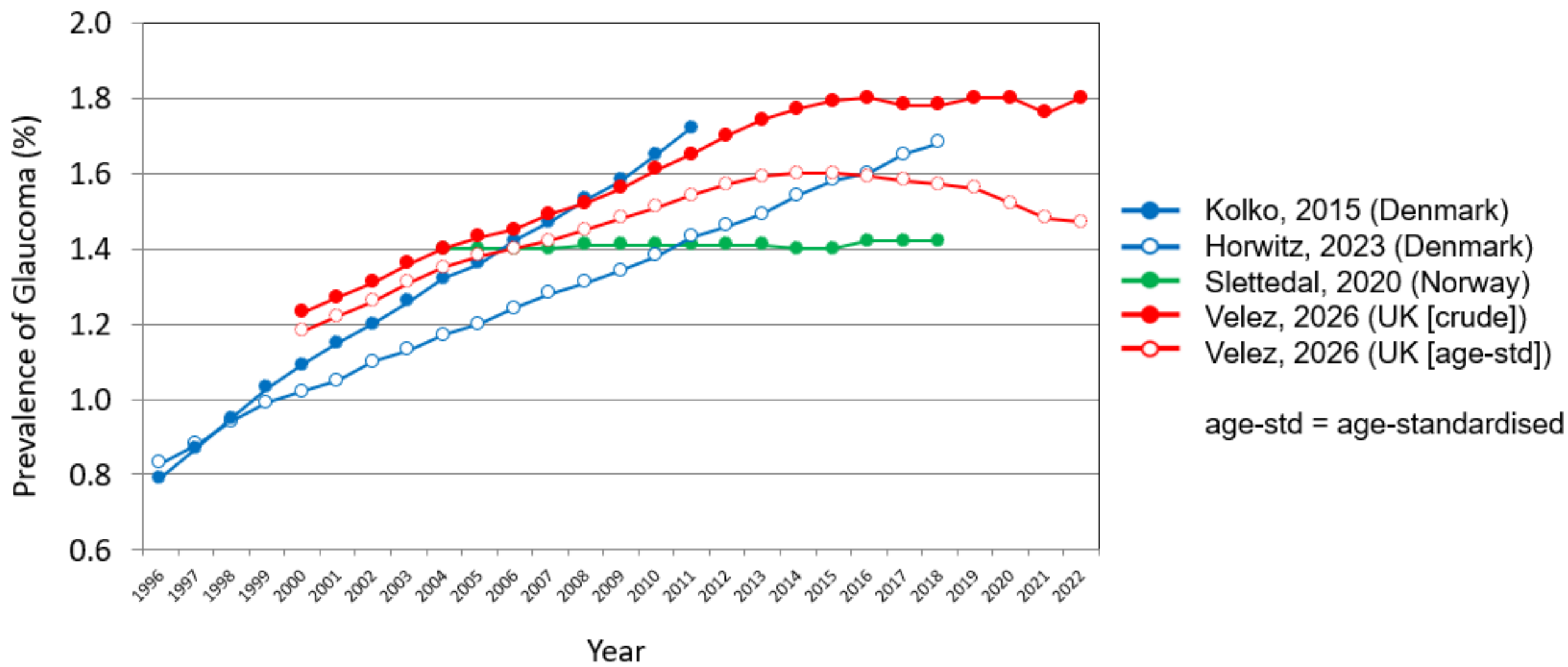

Supplement: Supplementary file 4 [file pone.0354463.s004.pdf]
