# Supplementary material for: The incidence rates and prevalence of treated glaucoma in the United Kingdom, 2000–2022
Source: PLoS One. 2026 Sep 3;21(9):e0354463. doi: 10.1371/journal.pone.0354463 (PMC13541118; doi:10.1371/journal.pone.0354463)

S2 Fig. Incidence rates of glaucoma [per 100,000 person-years] from different studies.

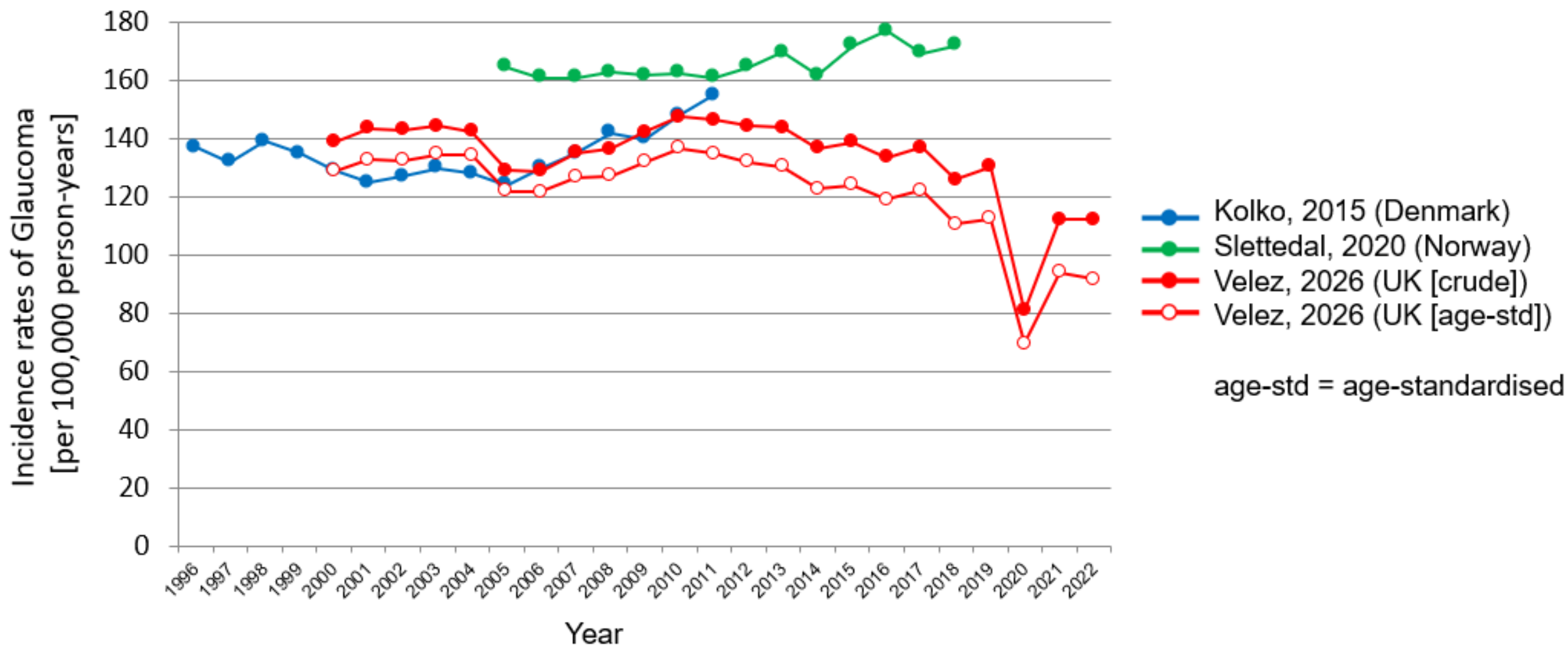

Supplement: Supplementary file 5 [file pone.0354463.s005.pdf]
