# Supplementary material for: The incidence rates and prevalence of treated glaucoma in the United Kingdom, 2000–2022
Source: PLoS One. 2026 Sep 3;21(9):e0354463. doi: 10.1371/journal.pone.0354463 (PMC13541118; doi:10.1371/journal.pone.0354463)
Supplement: Supplementary file 6 [file pone.0354463.s006.docx]

To

**Doctor Ryo Asaoka**
**Academic Editor**

**Public Library of Science (PLOS)**

Basel, April 21st, 2026.

**Velez-Nandayapa et al.: Incidence rates and prevalence of treated glaucoma in the United Kingdom, 2000-2022**

Dear Doctor Asaoka,

We thank you and the reviewers for your careful review of our manuscript and for the thoughtful, constructive comments provided. We greatly appreciate the time and expertise you invested in reviewing our work; your suggestions have helped to improve the manuscript’s clarity, rigor, and presentation.

We have received all comments and have addressed each one in detail. In the revised submission we provide a point-by-point response to every reviewer comment, numbered to correspond with the reviewers’ original remarks. For ease of review, changes made in the manuscript are highlighted (or tracked) and the location of each change is indicated (section, page, and paragraph or line numbers as appropriate).

Our comments addressing your points are mentioned below:

**Comment 1. PLOS ONE's style requirements.** The authors would like to state that our manuscript meets PLOS ONE’s style requirements, including those for file naming.

**Comment 2. Statement about third-party data.** The authors would like to state that we have a statement in our manuscript titled **“Data Accessibility Statement”** (page 2, lines 25–28) which reads: “Due to official regulations by the data provider, sharing of analytical data is strictly prohibited. Any analytical data must be stored on a pre-specified and agreed-upon server with strict access control. Data can be requested via an official data request submitted to the Clinical Practice Research Datalink (CPRD).” We have changed **“Data Accessibility Statement”** to **“Data Availability Statement”** in the manuscript.

**Comment 3. The authors would like to clarify the commercial affiliation of Dr. Laura Eggenschwiler with Augenpraxis Kuessnacht.** Dr. Eggenschwiler is an ophthalmologist who works at Augenpraxis Kuessnacht, Kuessnacht, Switzerland. The authors confirm that this commercial affiliation did not alter our adherence to all PLOS ONE policies. We will provide the following changes:

I. An amendment to the **Funding Statement** declaring that this commercial affiliation did not play a role in the study design, data collection and analysis, decision to publish, or preparation of the manuscript, and that it only provided financial support in the form of authors' salaries and/or research materials.
II. An updated **Competing Interests Statement** declaring Dr. Laura Eggenschwiler’s commercial affiliation. Within the Competing Interests Statement, we will confirm that this affiliation did not alter our adherence to all PLOS ONE policies on sharing data and materials by including the following sentence: “This does not alter our adherence to PLOS ONE policies on sharing data and materials.”
III. We will include both an updated **Funding Statement** and **Competing Interests Statement** in our **cover letter**.

**Comment 4. Ethics statement.** Per your instruction, we have moved the **Ethics statement** to the Methods section (page 8, lines 168 to 177).

**Comment 5. References.** We reviewed the entire reference list and we confirm that none of the cited articles have been retracted, and that our references comply with PLOS ONE requirements.

**Comment 6. Reviewer #1 questions:**

a) **Who confirmed the diagnosis of glaucoma in these patients and where were the diagnosis made; at secondary of tertiary eye centres in UK?**

Answer. Under UK practice, General Practitioners and optometrists perform preliminary evaluations and, when glaucoma is suspected, refer patients to ophthalmologists in secondary (district or community ophthalmology clinics) or tertiary centers (hospital-based specialist units) for diagnostic confirmation and treatment.

b) **What is the magnitude of untreated or undiagnosed glaucoma in UK? This will help in assessing the usefulness of this proxy data.**

Answer. In a previous analysis performed by our group covering the period from 2000 to 2017, we used two different definitions of glaucoma: glaucoma diagnosis plus two glaucoma prescriptions and/or glaucoma surgery versus only glaucoma diagnosis. We observed 21.6% more patients who had a diagnosis of glaucoma but were not treated. We observed that this gap was highest in London at 32.4%, followed by England (excluding London) 22.0%, Wales 19.6%, Scotland 17.4%, and lowest in Northern Ireland at 15.6%.

This previous analysis suggests that around 21.6% of patients with an incident glaucoma diagnosis are not treated pharmacologically and/or with glaucoma surgery in the UK.

To address the question of reviewer 1 regarding the magnitude of undiagnosed glaucoma, we compared our results with published literature. We report an overall age-standardised prevalence of glaucoma of 1.46%, with prevalences of 1.72% among those aged 40–79 years and 9.40% among those aged ≥80 years. A meta-analysis by Tham et al. (2014) estimated the combined prevalence of POAG and PACG at 3.54% in people aged 40–80 years (2.93% in Europe); our estimate is roughly half of that reported by Tham et al. Another meta-analysis performed by Kapetanakis et al. (2016) reported an overall POAG prevalence of 2.2%. An earlier study in Scandinavian countries by Goldschmidt et al. (1989), using prescriptions as a proxy for glaucoma, reported overall prevalences of 0.33%–0.92% across countries and 1.44%–3.15% in those over 40. A study from Iceland by Viggósson et al. (1986) reported an overall prevalence of 0.83%, 3.53% in those over 50, and 10.75% in those over 80- findings broadly comparable to ours.

To the best of our knowledge, our work represents the largest nationwide study of incidence rates and prevalence of glaucoma in the UK. We acknowledge that further work is needed on glaucoma diagnosis and treatment, and we hope our findings help quantify the magnitude of treated glaucoma nationally. We also hope that targeted screening for people with established risk factors will increase in coming years, leading to improved detection and management and, potentially, reduced blindness from glaucoma. Glaucoma remains a growing burden on the UK National Health Service.

c) **Ethnicity and or race is recognised risk factor for glaucoma occurrence and its progression. UK is a very cosmopolitan region. Why did the authors use 'region' in this study?**

Answer. We agree that race/ethnicity is an important risk factor for glaucoma. However, in this study we aim to provide information on questions like access to health care and more social aspects. We therefore used “geographic region” rather than “ethnicity”.
Ethnicity recording in routine primary-care data is often incomplete and heterogeneous, which can introduce bias and reduce statistical power for subgroup analyses; by contrast, region is consistently coded and reliably captures place of care and population context. Region also reflects important health-service factors (referral patterns, access to secondary/tertiary eye services, and local service provision) that influence the ascertainment and treated prevalence of glaucoma - the primary focus of our study.

Our results suggest that the differences observed across regions may be explained by varying access to healthcare.

We agree with the reviewer that it would be interesting to analyse the impact of ethnicity on our results. However, this was not the focus of our initial study and thus we did not include a variable for ethnicity in our dataset.

**Comment 7. Reviewer #2 question:**

a) **Excellent work on translational medicine on a topic which needs extensive knowledge improvement. Maybe genetic testing will be more easily available at affordable costs to make this more useful in clinical medicine.**

Answer. Thank you for your positive feedback and insightful comment. We agree that increasing availability and affordability of genetic testing may enhance its future utility in clinical medicine. However, at present, the clinical role of genetic testing in glaucoma remains limited. While genetic testing is well established and clinically useful for rare forms such as congenital or juvenile‑onset glaucoma, most adult‑onset glaucoma cases are genetically complex and polygenic. In these cases, currently available genetic tests or polygenic risk scores do not provide sufficient diagnostic accuracy to replace established clinical assessments. Therefore, despite anticipated reductions in cost and broader access, further validation and demonstration of clinical utility are required before genetic testing can be routinely integrated into the diagnosis of common forms of glaucoma.

Thank you again for your thoughtful review and helpful suggestions.

Sincerely,

Luis Velez-Nandayapa, MD, MSc, PhD candidate (corresponding author)

luis.velez@unibas.ch
