# Supplementary material for: The incidence rates and prevalence of treated glaucoma in the United Kingdom, 2000–2022
Source: PLoS One. 2026 Sep 3;21(9):e0354463. doi: 10.1371/journal.pone.0354463 (PMC13541118; doi:10.1371/journal.pone.0354463)
Supplement: Peer review history

# Peer review: Decision Letter 0

**DOI:** 10.1371/journal.pone.0354463.r001

**Authors:** Ryo Asaoka

5 Mar 2026

Dear Dr. Velez-Nandayapa,

Thank you for submitting your manuscript to PLOS ONE. After careful consideration, we feel that it has merit but does not fully meet PLOS ONE’s publication criteria as it currently stands. Therefore, we invite you to submit a revised version of the manuscript that addresses the points raised during the review process.

Please submit your revised manuscript by Apr 19 2026 11:59PM. If you will need more time than this to complete your revisions, please reply to this message or contact the journal office at plosone@plos.org. When you're ready to submit your revision, log on to https://www.editorialmanager.com/pone/ and select the 'Submissions Needing Revision' folder to locate your manuscript file.

- A letter that responds to each point raised by the academic editor and reviewer(s). You should upload this letter as a separate file labeled 'Response to Reviewers'.
- A marked-up copy of your manuscript that highlights changes made to the original version. You should upload this as a separate file labeled 'Revised Manuscript with Track Changes'.
- An unmarked version of your revised paper without tracked changes. You should upload this as a separate file labeled 'Manuscript'.

If applicable, we recommend that you deposit your laboratory protocols in protocols.io to enhance the reproducibility of your results. Protocols.io assigns your protocol its own identifier (DOI) so that it can be cited independently in the future. For instructions see: https://journals.plos.org/plosone/s/submission-guidelines#loc-laboratory-protocols. Additionally, PLOS ONE offers an option for publishing peer-reviewed Lab Protocol articles, which describe protocols hosted on protocols.io. Read more information on sharing protocols at https://plos.org/protocols?utm_medium=editorial-email&utm_source=authorletters&utm_campaign=protocols.

We look forward to receiving your revised manuscript.

Kind regards,

Ryo Asaoka

Academic Editor

PLOS One

Journal Requirements:

When submitting your revision, we need you to address these additional requirements.

1. Please ensure that your manuscript meets PLOS ONE's style requirements, including those for file naming. The PLOS ONE style templates can be found at

https://journals.plos.org/plosone/s/file?id=wjVg/PLOSOne_formatting_sample_main_body.pdf and https://journals.plos.org/plosone/s/file?id=ba62/PLOSOne_formatting_sample_title_authors_affiliations.pdf

2. For studies involving third-party data, we encourage authors to share any data specific to their analyses that they can legally distribute. PLOS recognizes, however, that authors may be using third-party data they do not have the rights to share. When third-party data cannot be publicly shared, authors must provide all information necessary for interested researchers to apply to gain access to the data. (https://journals.plos.org/plosone/s/data-availability#loc-acceptable-data-access-restrictions)

For any third-party data that the authors cannot legally distribute, they should include the following information in their Data Availability Statement upon submission:

1) A description of the data set and the third-party source

2) If applicable, verification of permission to use the data set

3) Confirmation of whether the authors received any special privileges in accessing the data that other researchers would not have

4) All necessary contact information others would need to apply to gain access to the data

3. Thank you for stating the following in the Competing Interests section:

[The authors have declared that no competing interests exist.].

We note that one or more of the authors are employed by a commercial company: Augenpraxis Küssnacht.

1. Please provide an amended Funding Statement declaring this commercial affiliation, as well as a statement regarding the Role of Funders in your study. If the funding organization did not play a role in the study design, data collection and analysis, decision to publish, or preparation of the manuscript and only provided financial support in the form of authors' salaries and/or research materials, please review your statements relating to the author contributions, and ensure you have specifically and accurately indicated the role(s) that these authors had in your study. You can update author roles in the Author Contributions section of the online submission form.

Please also include the following statement within your amended Funding Statement.

“The funder provided support in the form of salaries for authors [insert relevant initials], but did not have any additional role in the study design, data collection and analysis, decision to publish, or preparation of the manuscript. The specific roles of these authors are articulated in the ‘author contributions’ section.”

If your commercial affiliation did play a role in your study, please state and explain this role within your updated Funding Statement.

2. Please also provide an updated Competing Interests Statement declaring this commercial affiliation along with any other relevant declarations relating to employment, consultancy, patents, products in development, or marketed products, etc.

Within your Competing Interests Statement, please confirm that this commercial affiliation does not alter your adherence to all PLOS ONE policies on sharing data and materials by including the following statement: "This does not alter our adherence to PLOS ONE policies on sharing data and materials.” (as detailed online in our guide for authors http://journals.plos.org/plosone/s/competing-interests). If this adherence statement is not accurate and there are restrictions on sharing of data and/or materials, please state these. Please note that we cannot proceed with consideration of your article until this information has been declared.

Please include both an updated Funding Statement and Competing Interests Statement in your cover letter. We will change the online submission form on your behalf.

4. Your ethics statement should only appear in the Methods section of your manuscript. If your ethics statement is written in any section besides the Methods, please move it to the Methods section and delete it from any other section. Please ensure that your ethics statement is included in your manuscript, as the ethics statement entered into the online submission form will not be published alongside your manuscript.

If the reviewer comments include a recommendation to cite specific previously published works, please review and evaluate these publications to determine whether they are relevant and should be cited. There is no requirement to cite these works unless the editor has indicated otherwise.

Please review your reference list to ensure that it is complete and correct. If you have cited papers that have been retracted, please include the rationale for doing so in the manuscript text, or remove these references and replace them with relevant current references. Any changes to the reference list should be mentioned in the rebuttal letter that accompanies your revised manuscript. If you need to cite a retracted article, indicate the article’s retracted status in the References list and also include a citation and full reference for the retraction notice.

Additional Editor Comments:

Both reviewers gave positive feedbacks, however please respond to the points raised by the reviewers.

[Note: HTML markup is below. Please do not edit.]

Reviewers' comments:

Reviewer's Responses to Questions

**Comments to the Author**

1. Is the manuscript technically sound, and do the data support the conclusions?

Reviewer #1: Yes

Reviewer #2: Yes

**********

2. Has the statistical analysis been performed appropriately and rigorously?

Reviewer #1: Yes

Reviewer #2: I Don't Know

**********

3. Have the authors made all data underlying the findings in their manuscript fully available?

The PLOS Data policy

Reviewer #1: Yes

Reviewer #2: Yes

**********

4. Is the manuscript presented in an intelligible fashion and written in standard English?

Reviewer #1: Yes

Reviewer #2: Yes

**********

Reviewer #1: Glaucoma is an important cause of irreversible vision loss worldwide. The use of glaucoma treatment (medical or surgical) a proxy for estimating glaucoma prevalence is noted. I want make the following comments:

a. Who confirmed the diagnosis of glaucoma in these patients and where were the diagnosis made; at secondary of tertiary eye centres in UK?

b. what is the magnitude of untreated or undiagnosed glaucoma in UK? This will help in assessing the usefulness of this proxy data.

c. Ethnicity and or race is recognised risk factor for glaucoma occurrence and its progression. UK is a very cosmopolitan region. Why did the authors use 'region' in this study?

Reviewer #2: Excellent work on translational medicine on a topic which needs extensive knowledge improvement. Maybe genetic testing will be more easily available at affordable costs to make this more useful in clinical medicine.

**********

what does this mean?). If published, this will include your full peer review and any attached files.

If you choose “no”, your identity will remain anonymous but your review may still be made public.

**Do you want your identity to be public for this peer review?**  For information about this choice, including consent withdrawal, please see our Privacy Policy

Reviewer #1: No

Reviewer #2: No

**********

[NOTE: If reviewer comments were submitted as an attachment file, they will be attached to this email and accessible via the submission site. Please log into your account, locate the manuscript record, and check for the action link "View Attachments". If this link does not appear, there are no attachment files.]

To ensure your figures meet our technical requirements, please review our figure guidelines: https://journals.plos.org/plosone/s/figures

You may also use PLOS’s free figure tool, NAAS, to help you prepare publication quality figures: https://journals.plos.org/plosone/s/figures#loc-tools-for-figure-preparation.

NAAS will assess whether your figures meet our technical requirements by comparing each figure against our figure specifications.

# Author comment: Author response to Decision Letter 1

**DOI:** 10.1371/journal.pone.0354463.r002

23 Jun 2026

Doctor Ryo Asaoka

Academic Editor

Public Library of Science (PLOS)

Basel, April 21st, 2026.

Velez-Nandayapa et al.: Incidence rates and prevalence of treated glaucoma in the United Kingdom, 2000-2022

Dear Doctor Asaoka,

We thank you and the reviewers for your careful review of our manuscript and for the thoughtful, constructive comments provided. We greatly appreciate the time and expertise you invested in reviewing our work; your suggestions have helped to improve the manuscript’s clarity, rigor, and presentation.

We have received all comments and have addressed each one in detail. In the revised submission we provide a point-by-point response to every reviewer comment, numbered to correspond with the reviewers’ original remarks. For ease of review, changes made in the manuscript are highlighted (or tracked) and the location of each change is indicated (section, page, and paragraph or line numbers as appropriate).

Our comments addressing your points are mentioned below:

Comment 1. PLOS ONE's style requirements. The authors would like to state that our manuscript meets PLOS ONE’s style requirements, including those for file naming.

Comment 2. Statement about third-party data. The authors would like to state that we have a statement in our manuscript titled “Data Accessibility Statement” (page 2, lines 25–28) which reads: “Due to official regulations by the data provider, sharing of analytical data is strictly prohibited. Any analytical data must be stored on a pre-specified and agreed-upon server with strict access control. Data can be requested via an official data request submitted to the Clinical Practice Research Datalink (CPRD).” We have changed “Data Accessibility Statement” to “Data Availability Statement” in the manuscript.

Comment 3. The authors would like to clarify the commercial affiliation of Dr. Laura Eggenschwiler with Augenpraxis Kuessnacht. Dr. Eggenschwiler is an ophthalmologist who works at Augenpraxis Kuessnacht, Kuessnacht, Switzerland. The authors confirm that this commercial affiliation did not alter our adherence to all PLOS ONE policies. We will provide the following changes:

I. An amendment to the Funding Statement declaring that this commercial affiliation did not play a role in the study design, data collection and analysis, decision to publish, or preparation of the manuscript, and that it only provided financial support in the form of authors' salaries and/or research materials.

II. An updated Competing Interests Statement declaring Dr. Laura Eggenschwiler’s commercial affiliation. Within the Competing Interests Statement, we will confirm that this affiliation did not alter our adherence to all PLOS ONE policies on sharing data and materials by including the following sentence: “This does not alter our adherence to PLOS ONE policies on sharing data and materials.”

III. We will include both an updated Funding Statement and Competing Interests Statement in our cover letter.

Comment 4. Ethics statement. Per your instruction, we have moved the Ethics statement to the Methods section (page 8, lines 168 to 177).

Comment 5. References. We reviewed the entire reference list and we confirm that none of the cited articles have been retracted, and that our references comply with PLOS ONE requirements.

Comment 6. Reviewer #1 questions:

a) Who confirmed the diagnosis of glaucoma in these patients and where were the diagnosis made; at secondary of tertiary eye centres in UK?

Answer. Under UK practice, General Practitioners and optometrists perform preliminary evaluations and, when glaucoma is suspected, refer patients to ophthalmologists in secondary (district or community ophthalmology clinics) or tertiary centers (hospital-based specialist units) for diagnostic confirmation and treatment.

b) What is the magnitude of untreated or undiagnosed glaucoma in UK? This will help in assessing the usefulness of this proxy data.

Answer. In a previous analysis performed by our group covering the period from 2000 to 2017, we used two different definitions of glaucoma: glaucoma diagnosis plus two glaucoma prescriptions and/or glaucoma surgery versus only glaucoma diagnosis. We observed 21.6% more patients who had a diagnosis of glaucoma but were not treated. We observed that this gap was highest in London at 32.4%, followed by England (excluding London) 22.0%, Wales 19.6%, Scotland 17.4%, and lowest in Northern Ireland at 15.6%.

This previous analysis suggests that around 21.6% of patients with an incident glaucoma diagnosis are not treated pharmacologically and/or with glaucoma surgery in the UK.

To address the question of reviewer 1 regarding the magnitude of undiagnosed glaucoma, we compared our results with published literature. We report an overall age-standardised prevalence of glaucoma of 1.46%, with prevalences of 1.72% among those aged 40–79 years and 9.40% among those aged ≥80 years. A meta-analysis by Tham et al. (2014) estimated the combined prevalence of POAG and PACG at 3.54% in people aged 40–80 years (2.93% in Europe); our estimate is roughly half of that reported by Tham et al. Another meta-analysis performed by Kapetanakis et al. (2016) reported an overall POAG prevalence of 2.2%. An earlier study in Scandinavian countries by Goldschmidt et al. (1989), using prescriptions as a proxy for glaucoma, reported overall prevalences of 0.33%–0.92% across countries and 1.44%–3.15% in those over 40. A study from Iceland by Viggósson et al. (1986) reported an overall prevalence of 0.83%, 3.53% in those over 50, and 10.75% in those over 80- findings broadly comparable to ours.

To the best of our knowledge, our work represents the largest nationwide study of incidence rates and prevalence of glaucoma in the UK. We acknowledge that further work is needed on glaucoma diagnosis and treatment, and we hope our findings help quantify the magnitude of treated glaucoma nationally. We also hope that targeted screening for people with established risk factors will increase in coming years, leading to improved detection and management and, potentially, reduced blindness from glaucoma. Glaucoma remains a growing burden on the UK National Health Service.

c) Ethnicity and or race is recognised risk factor for glaucoma occurrence and its progression. UK is a very cosmopolitan region. Why did the authors use 'region' in this study?

Answer. We agree that race/ethnicity is an important risk factor for glaucoma. However, in this study we aim to provide information on questions like access to health care and more social aspects. We therefore used “geographic region” rather than “ethnicity”.

Ethnicity recording in routine primary-care data is often incomplete and heterogeneous, which can introduce bias and reduce statistical power for subgroup analyses; by contrast, region is consistently coded and reliably captures place of care and population context. Region also reflects important health-service factors (referral patterns, access to secondary/tertiary eye services, and local service provision) that influence the ascertainment and treated prevalence of glaucoma - the primary focus of our study.

Our results suggest that the differences observed across regions may be explained by varying access to healthcare.

We agree with the reviewer that it would be interesting to analyse the impact of ethnicity on our results. However, this was not the focus of our initial study and thus we did not include a variable for ethnicity in our dataset.

Comment 7. Reviewer #2 question:

a) Excellent work on translational medicine on a topic which needs extensive knowledge improvement. Maybe genetic testing will be more easily available at affordable costs to make this more useful in clinical medicine.

Answer. Thank you for your positive feedback and insightful comment. We agree that increasing availability and affordability of genetic testing may enhance its future utility in clinical medicine. However, at present, the clinical role of genetic testing in glaucoma remains limited. While genetic testing is well established and clinically useful for rare forms such as congenital or juvenile‑onset glaucoma, most adult‑onset glaucoma cases are genetically complex and polygenic. In these cases, currently available genetic tests or polygenic risk scores do not provide sufficient diagnostic accuracy to replace established clinical assessments. Therefore, despite anticipated reductions in cost and broader access, further validation and demonstration of clinical utility are required before genetic testing can be routinely integrated into the diagnosis of common forms of glaucoma.

Thank you again for your thoughtful review and helpful suggestions.

Sincerely,

Luis Velez-Nandayapa, MD, MSc, PhD candidate (corresponding author)

luis.velez@unibas.ch

Attachment Submitted filename: Response Reviewers_Glaucoma_Prev_IR_Velez-Nandayapa_21Apr26.docx

# Peer review: Decision Letter 1

**DOI:** 10.1371/journal.pone.0354463.r003

**Authors:** Ryo Asaoka

9 Jul 2026

The incidence rates and prevalence of treated glaucoma in the United Kingdom, 2000-2022

PONE-D-25-65736R1

Dear Dr. Velez-Nandayapa,

We’re pleased to inform you that your manuscript has been judged scientifically suitable for publication and will be formally accepted for publication once it meets all outstanding technical requirements.

Within one week, you’ll receive an e-mail detailing the required amendments. When these have been addressed, you’ll receive a formal acceptance letter and your manuscript will be scheduled for publication.

An invoice will be generated when your article is formally accepted. Please note, if your institution has a publishing partnership with PLOS and your article meets the relevant criteria, all or part of your publication costs will be covered. Please make sure your user information is up-to-date by logging into Editorial Manager at Editorial Manager® and clicking the ‘Update My Information' link at the top of the page. For questions related to billing, please contact billing support.

If your institution or institutions have a press office, please notify them about your upcoming paper to help maximize its impact. If they’ll be preparing press materials, please inform our press team as soon as possible -- no later than 48 hours after receiving the formal acceptance. Your manuscript will remain under strict press embargo until 2 pm Eastern Time on the date of publication. For more information, please contact onepress@plos.org.

Kind regards,

Ryo Asaoka

Academic Editor

PLOS One

Additional Editor Comments (optional):

The authors successfully handled the concerns raised by the reviewers.

Reviewers' comments:

Reviewer's Responses to Questions

**Comments to the Author**

Reviewer #1: All comments have been addressed

**********

2. Is the manuscript technically sound, and do the data support the conclusions?

Reviewer #1: Yes

**********

3. Has the statistical analysis been performed appropriately and rigorously?

Reviewer #1: Yes

**********

4. Have the authors made all data underlying the findings in their manuscript fully available?

The PLOS Data policy

Reviewer #1: Yes

**********

5. Is the manuscript presented in an intelligible fashion and written in standard English?

Reviewer #1: Yes

**********

Reviewer #1: The authors have adequately responded to questions raised by me. Diagnosis of glaucoma is complex thus the need for clear information on the standard of care for glaucoma diagnosis in UK.

**********

what does this mean?). If published, this will include your full peer review and any attached files.

If you choose “no”, your identity will remain anonymous but your review may still be made public.

**Do you want your identity to be public for this peer review?**  For information about this choice, including consent withdrawal, please see our Privacy Policy

Reviewer #1: No

**********

# Editor report: Acceptance letter

**DOI:** 10.1371/journal.pone.0354463.r004

**Authors:** Ryo Asaoka

PONE-D-25-65736R1

PLOS One

Dear Dr. Velez-Nandayapa,

I'm pleased to inform you that your manuscript has been deemed suitable for publication in PLOS One. Congratulations! Your manuscript is now being handed over to our production team.

At this stage, our production department will prepare your paper for publication. This includes ensuring the following:

* All references, tables, and figures are properly cited

* All relevant supporting information is included in the manuscript submission,

* There are no issues that prevent the paper from being properly typeset

You will receive further instructions from the production team, including instructions on how to review your proof when it is ready. Please keep in mind that we are working through a large volume of accepted articles, so please give us a few days to review your paper and let you know the next and final steps.

Lastly, if your institution or institutions have a press office, please let them know about your upcoming paper now to help maximize its impact. If they'll be preparing press materials, please inform our press team within the next 48 hours. Your manuscript will remain under strict press embargo until 2 pm Eastern Time on the date of publication. For more information, please contact onepress@plos.org.

You will receive an invoice from PLOS for your publication fee after your manuscript has reached the completed accept phase. If you receive an email requesting payment before acceptance or for any other service, this may be a phishing scheme. Learn how to identify phishing emails and protect your accounts at https://explore.plos.org/phishing.

If we can help with anything else, please email us at customercare@plos.org.

Thank you for submitting your work to PLOS One and supporting open access.

Kind regards,

PLOS One Editorial Office Staff

on behalf of

Dr. Ryo Asaoka

Academic Editor

PLOS One
